# Molecular insights into the persistence and co-occurrence of two different carbapenem-resistant *Pseudomonas aeruginosa* lineages within a hospital setting

**DOI:** 10.1128/spectrum.00433-25

**Published:** 2025-09-17

**Authors:** Xinran Li, Kaiying Wang, Jiali Chen, Zixuan Chen, Jinhui Li, Peng Li, Xiong Liu, Suling Liu

**Affiliations:** 1 Chinese PLA Center for Disease Control and Prevention 666583 , Beijing, China; 2 China Medical University, School of Public Health 540411 , Shenyang, China; 3 Nankai University, School of Medicine 481107, Tianjin, China; 4 Northern Theater Command Center for Disease Control and Prevention 604347 , Shenyang, China; 5 Department of Laboratory Medicine, Guangdong Provincial People’s Hospital (Guangdong Academy of Medical Sciences), Southern Medical University, Guangzhou, China; Istituto Dermatologico San Gallicano, Rome, Italy

**Keywords:** *Pseudomonas aeruginosa*, carbapenem resistant, molecular epidemiology

## Abstract

Carbapenem-resistant *Pseudomonas aeruginosa* (CRPA) represents a critical-priority pathogen capable of causing life-threatening, multidrug-resistant infections. We performed susceptibility testing, whole-genome sequencing, and bioinformatic analyses on 137 CRPA isolates from a Guangdong hospital. We found that the major specimen types were respiratory specimens (57/137, 41.6%) and bronchoalveolar lavage (42/137, 30.7%). All isolates were carbapenem-resistant but had low resistance to polymyxin B (0.7%, 1/137). IncP-6-positive isolates exhibited ≥2- to 32-fold higher resistance to 9/12 antibiotics (*P* < 0.05), with no difference to imipenem and meropenem. Fifty-four sequence types and 11 O-serogroups were identified, with ST1971 (6.6%) and O11 (29.9%) being predominant. Temporal and spatial patterns suggest persistent co-occurrence of clade 1 and clade 2 isolates, indicating potential nosocomial outbreak and clonal transmission.

**IMPORTANCE:**

The prevalence of carbapenem-resistant *Pseudomonas aeruginosa* (CRPA) has increased rapidly in recent years, yet few genetic and epidemiological studies on CRPA isolates have been performed. We performed susceptibility testing, whole-genome sequencing, and bioinformatic analyses on hospital isolates to investigate their resistance profiles and molecular epidemiology. These findings may offer new insights for developing effective global strategies to control CRPA and reduce untreatable infections in clinical settings.

## INTRODUCTION

*Pseudomonas aeruginosa*, a gram-negative opportunistic pathogen, is commonly found in soil, water, insects, and plants, as well as being prevalent in hospital settings (1, 2). It is a significant cause of healthcare-associated infections, being particularly problematic in intensive care units (3). *P. aeruginosa* causes a variety of hospital infections, including infections of the respiratory tract (4), blood (5), urinary tract, abdominal cavity, skin (6), surgery, and transplantation (7). The bacterium shows a high mutation rate with a complex antibiotic resistance mechanism (8).

Carbapenem antibiotics are crucial in treating various bacterial infections due to their broad spectrum, potent antibacterial activity, and high stability against extended-spectrum β-lactamases (ESBLs) (9). However, their extensive use against multidrug-resistant *P. aeruginosa* infections has driven the emergence and spread of carbapenem-resistant *P. aeruginosa* (CRPA) (10). The widespread prevalence of CRPA has become a formidable problem, posing significant challenges in clinical anti-infective therapy, as it complicates the selection of effective antibiotics and prolongs patient recovery.

CRPA has rapidly increased in prevalence in recent years and now represents a significant threat to global public health. However, few genetic and molecular epidemiology studies of CRPA isolates have been performed in China. Here, we conducted a genome-based survey of the prevalence of CRPA isolates collected from various departments. This study aimed to investigate the resistance profiles and molecular epidemiology of CRPA isolates from a hospital in Guangdong, China, focusing on resistance mechanisms, phylogenomic analysis, and transmission dynamics to help control its spread in healthcare settings.

## MATERIALS AND METHODS

### CRPA isolates and clinical data collection

A total of 137 CRPA isolates were collected from clinical samples at Guangdong Provincial People’s Hospital between 1 January 2022 and 26 July 2023. Clinical specimen types were categorized based on the 2020 surveillance protocol of CHINET (11): bronchoalveolar lavage, respiratory specimens, wound pus, blood, urine, sterile body fluids, catheter, and others. Table S1 provides more detailed source categories and metadata for each isolate.

### Antibiotic susceptibility testing

The antimicrobial susceptibility testing was performed on all 197 isolates using the VITEK2 COMPACT system (bioMérieux, France) in combination with the Kirby-Bauer (KB) method. Fourteen antibiotics were selected for this study, including amikacin, gentamicin, tobramycin, aztreonam, ceftazidime, cefepime, ciprofloxacin, levofloxacin, imipenem, meropenem, piperacillin, piperacillin/tazobactam, cefoperazone/sulbactam, and polymixin B. *P. aeruginosa* ATCC 27853 and *Escherichia coli* ATCC 25922 were used as control isolates. Antibiotic susceptibility testing results were interpreted according to the Clinical and Laboratory Standards Institute 2022 (12). Carbapenem resistance (CR) was defined as resistance to either imipenem or meropenem.

### Bioinformatics analyses

Genomic DNA from cultured bacteria was extracted using a DNA extraction kit (Qiagen, Germany). Then, whole-genome sequencing was performed on the Illumina NovaSeq 6000 platform at Novogene Co., Ltd. The quality of sequencing reads was evaluated by FastQC v0.11.9 (13). Low-quality reads and adaptors were filtered out and removed by Trimmomatic v0.39 (14). Clean data were assembled by Spades v3.13.0 (15). Then the quality of the acquired genome was assessed by Quast v5.2.0 (16) and CheckM v1.2.2 (17). Genomes were annotated by Prokka v1.14.6. The serotypes and the sequence types (STs) of CRPA isolates were determined using the PAst online tool (https://cge.food.dtu.dk/services/PAst/) and multilocus sequence typing (MLST) based on the *P. aeruginosa* PubMLST scheme (18), respectively. eBURST analysis was conducted to assess relationships between the different isolates on the basis of their STs and associated epidemiological data using PHYLOViZ (http://www.phyloviz.net/). Antibiotic resistance genes (ARGs), virulence genes, and plasmid replicon types were retrieved from the Resfinder (19), VFDB (20), and PlasmidFinder (21) databases using Abricate v1.0.1 (https://github.com/tseemann/abricate). Single-nucleotide polymorphisms (SNPs) were identified using Snippy v4.6.0, and the recombination regions were identified and excluded using ClonalFrameML v1.12. The maximum likelihood phylogenetic tree was inferred using IQ-TREE2 v2.2.7 (22) implemented with 1,000 bootstrap replicates. GTR + I + G4 was selected as the best evolutionary model using Modeltest-ng v0.1.7. The pairwise SNP distances among isolates were visualized in the supplemental material. A threshold of 25 SNPs was set to define transmission clusters, with genomes having fewer than 25 SNPs considered part of the same cluster.

### Statistical analysis

Differences in time distributions between clades were evaluated using the two-sample Kolmogorov-Smirnov test. The distribution of ARGs was described using the median and interquartile range (P25, P75). The Kruskal-Wallis *H* test was employed for non-parametric comparison of multiple groups. Fisher’s exact (two-tailed) or *χ*^2^ test was used for qualitative variables. *P* < 0.05 was regarded as statistically significant. Cluster significance was determined through Poisson-based log-likelihood ratio (LLR) tests, with significance thresholds set after Bonferroni adjustment (α = 0.05). The highest-LLR window was designated as the most likely cluster. The statistical analyses were performed using Python 3.11.

## RESULTS

### Clinical characteristics of CRPA isolates in clinical settings

**Fig 1:**
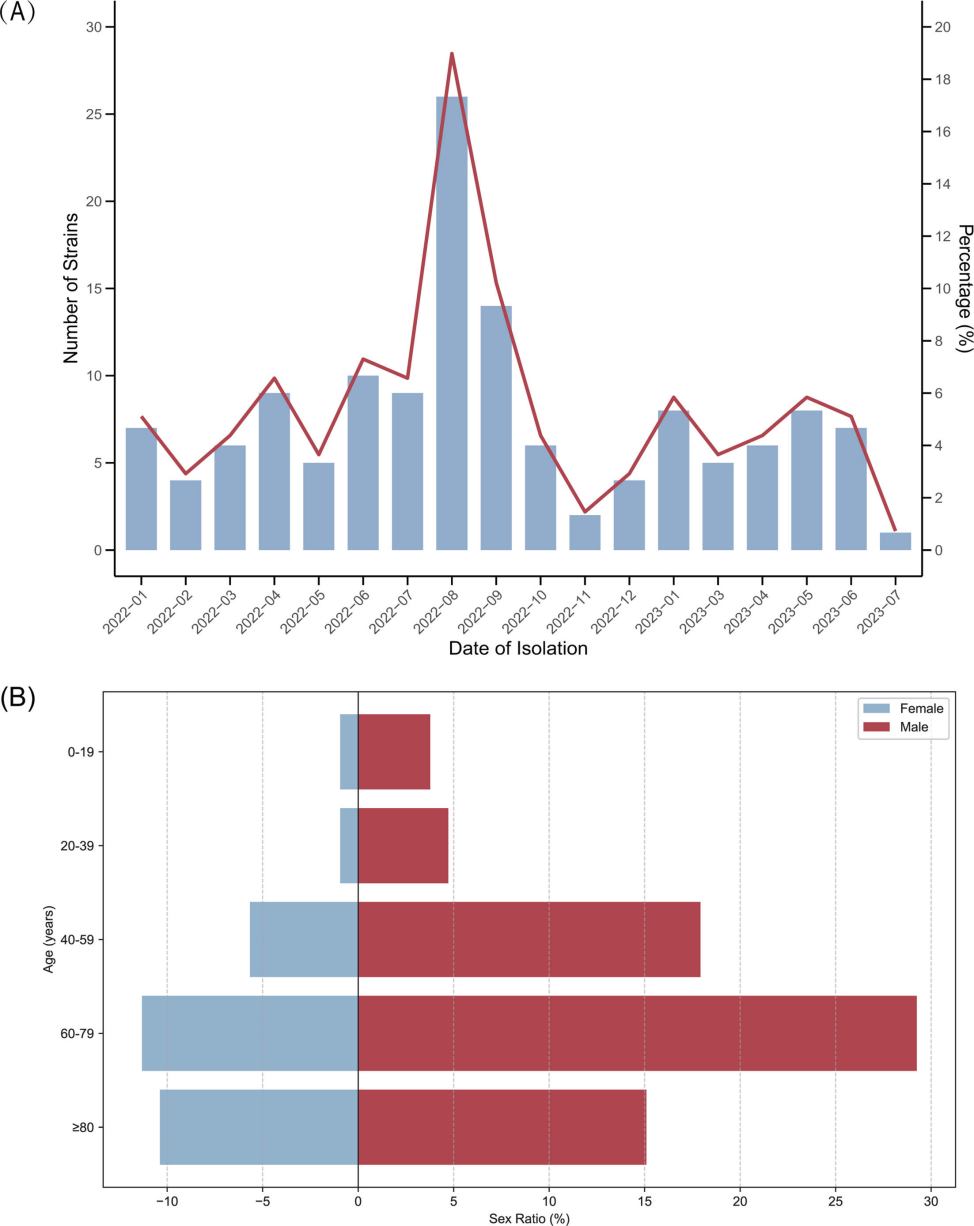
Clinical characteristics of patients with CRPA. (**A**) The distribution of CRPA isolates from January 2022 to July 2023. Bars indicate CRPA counts (primary *y*-axis). The red line shows monthly CRPA counts as a percentage of the total isolates (*n* = 137; secondary *y*-axis). (**B**) Age and sex distribution of patients. The *x*-axis represents the sex ratio, and the *y*-axis represents the age range. Only the first isolate per patient was included in the analysis.

From January 2022 to July 2023, a total of 137 CRPA isolates were collected from 106 patients (Fig. 1A). For 22 patients, 2–4 CRPA isolates were obtained from different time points or sample types (Table S4). As shown in Fig. 1B, the majority of patients were aged 60 years or older (70/106, 66.0%), and most were male (75/106, 70.8%). One hundred thirty-seven isolates were isolated from 26 departments, with the top three departments being geriatric intensive care unit (GICU; 25/137, 18.2%), intensive care unit (ICU; 22/137, 16.1%), and cardiac surgery ICU (21/137, 15.3%). Respiratory specimens were the most common source of isolates (41.6%), followed by bronchoalveolar lavage (30.7%) and wound pus (8.8%).

### Antimicrobial susceptibility testing

The antimicrobial susceptibilities of tested isolates to common antibacterial agents are shown in Table S2. All 137 isolates were identified as CRPA. The resistance rates to aminoglycoside antibiotics, including amikacin (11/137, 8.0%), gentamicin (12/137, 8.8%), and tobramycin (14/137, 10.2%), were consistently lower than the resistance rate to aztreonam, which exceeded 50%. Among the 137 CRPA isolates, 69 difficult-to-treat resistant *P. aeruginosa* (DTRPA) isolates were tested for resistance to the last-line drug polymyxin B, with only one isolate showing resistance (MIC ≥4 mg/L).

### Molecular typing analysis

**Fig 2:**
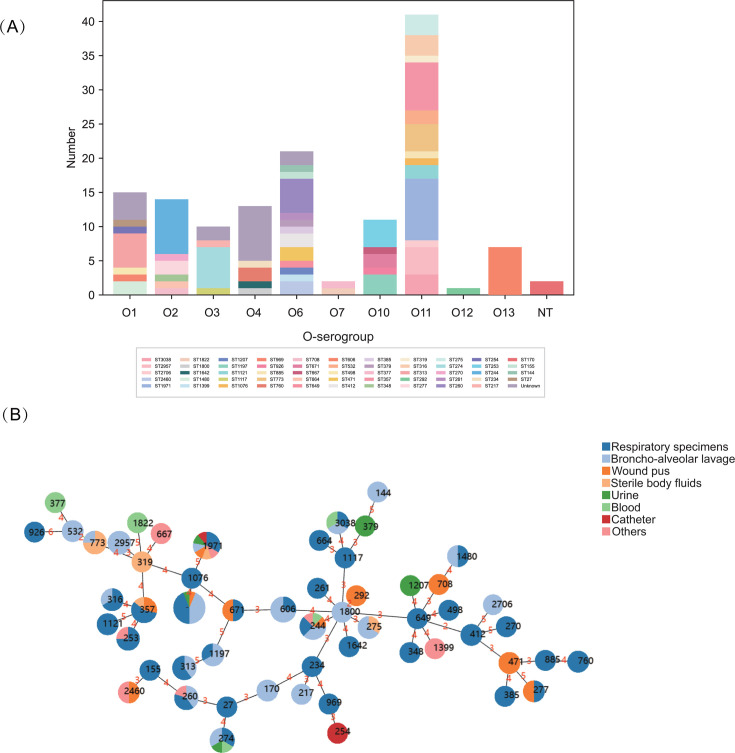
Distribution and relationship analysis of STs of 137 CRPA isolates. (**A**) The color-coded scheme illustrates the ST diversity across different O-serogroups. (**B**) The minimum spanning tree was constructed using PHYLOViZ Online (https://online.phyloviz.net/index). Each node (solid circle) represents an ST, with colors corresponding to sample sources. The node size is proportional to the number of isolates within each ST. The numbers on the connecting branches indicate the genetic distance between adjacent STs.

MLST analysis revealed that 121 isolates belonged to 54 STs, with the most commonly identified STs being ST1971 (9/137, 6.6%), ST244 (8/137, 5.8%), ST357 (7/137, 5.1%), and ST606 (7/137, 5.1%; Fig. 2A). The ST type of the remaining 16 isolates was not determined. Among all 137 CRPA isolates, 11 serogroups were identified, with O11 (29.9%, 41/137) being the most prevalent, followed by O6 (15.3%, 21/137) and O1 (10.9%, 15/137). Furthermore, clonal complex (CC) relationships were evidenced, including the following: CC1800 (ST1800 and ST292), CC649 (ST649 and ST412), and CC532 (ST532 and ST773; Fig. 2B). Out of a total of 54 ST types, 48 were found to be singletons. Distribution patterns for specimens and departments were discernible across different O-serogroups (Table 1). Bronchoalveolar lavage exhibited the highest O-serogroup diversity, with O1 (46.7%), O11 (31.7%), O3 (30.0%), and O4 (30.8%) being detected in distinct isolates. In contrast, isolates from catheter and sterile body fluids showed restricted O-serogroup associations. Notably, O13 and O4 were predominantly isolated from ICUs, while other serogroups were widely distributed across departments.

**TABLE 1:** Distributions of specimen and department among different O-serogroups (*n*, %)

Categories	O1 (*n* = 15)	O10 (*n* = 11)	O11 (*n* = 41)	O13 (*n* = 7)	O2 (*n* = 14)	O3 (*n* = 10)	O4 (*n* = 13)	O6 (*n* = 21)	Others^*a*^ (*n* = 5)
Specimen									
Blood	0 (0)	0 (0)	1 (2.4)	0 (0)	1 (7.1)	1 (10.0)	0 (0)	0 (0)	2 (40.0)
Bronchoalveolar lavage	7 (46.7)	2 (18.2)	13 (31.7)	5 (71.4)	3 (21.4)	3 (30.0)	4 (30.8)	3 (14.3)	2 (40.0)
Catheter	1 (6.7)	0 (0)	1 (2.4)	0 (0)	0 (0)	0 (0)	0 (0)	0 (0)	0 (0)
Others	0 (0)	2 (18.2)	1 (2.4)	0 (0)	1 (7.1)	0 (0)	0 (0)	3 (14.3)	0 (0)
Respiratory specimens	7 (46.7)	6 (54.5)	14 (34.1)	2 (28.6)	6 (42.9)	5 (50.0)	7 (53.8)	10 (47.6)	0 (0)
Sterile body fluids	0 (0)	0 (0)	7 (17.1)	0 (0)	0 (0)	0 (0)	0 (0)	0 (0)	0 (0)
Urine	0 (0)	0 (0)	1 (2.4)	0 (0)	0 (0)	1 (10.0)	1 (7.7)	2 (9.5)	0 (0)
Wound pus	0 (0)	1 (9.1)	3 (7.3)	0 (0)	3 (21.4)	0 (0)	1 (7.7)	3 (14.3)	1 (20.0)
Department									
Surgery	1 (6.7)	1 (9.1)	4 (9.8)	1 (14.3)	2 (14.3)	0 (0)	0 (0)	3 (14.3)	2 (40.0)
ICUs	10 (66.7)	7 (63.6)	24 (58.5)	6 (85.7)	6 (42.9)	6 (60.0)	11 (84.6)	12 (57.1)	3 (60.0)
Internal medicine	4 (26.7)	3 (27.3)	10 (24.4)	0 (0)	2 (14.3)	3 (30.0)	2 (15.4)	1 (4.8)	0 (0)
Others	0 (0)	0 (0)	3 (7.3)	0 (0)	4 (6.7)	1 (10.0)	0 (0)	5 (23.8)	0 (0)

^*a*^ Including non-typeable isolates, with two O7 and one O12 serotype identified.

### Analysis of ARGs and plasmid replicons

**Fig 3:**
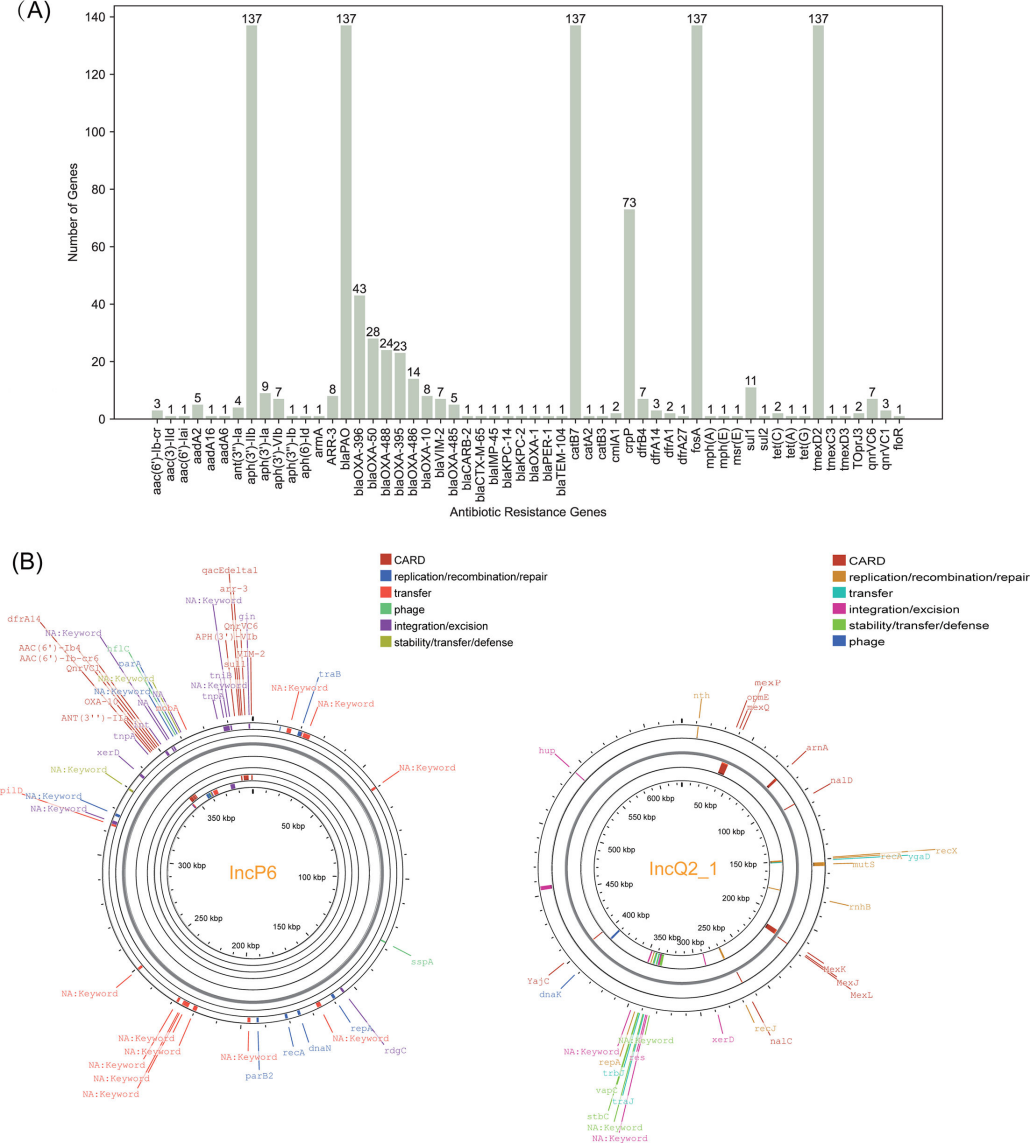
ARG profiles and plasmid architectures (IncP-6/IncQ2_1) in CRPA isolates. (**A**) The bar chart depicts the distribution of ARGs, with the *x*-axis representing the gene categories and the *y*-axis indicating the sequence counts. (**B**) Annotated genetic maps of the IncP-6 plasmid (ly147) and the IncQ2_1 plasmid (ly062) are presented.

The main resistance genes identified included *aph* (aminoglycosides), *bla* (beta-lactams), *cat* (chloramphenicol), *crpP* (ciprofloxacin), *fosA* (fosfomycin), and resistance-nodulation-division (RND) efflux pump genes (*tmexD2*, *tmexC3*, and *tmexD3*; Fig. 3A). The CRKP isolates carrying resistance genes exhibited M (P25, P75) values of 7 (6, 7) (Table 2). The number of resistance genes did not differ significantly among CRKP isolates from different specimen types (*P* = 0.72). The frequency of β-lactamase genes (*bla*_OXA-396_, *bla*_OXA-50_, *bla*_OXA-488_, *bla*_OXA-395_, *bla*_OXA-486_, and *bla*_OXA-485_) across different departments showed no statistically significant differences (*P* > 0.05; Table 3). The *bla*_OXA-10_ and *bla*_VIM-2_ genes were only detected in ICU isolates, while *bla*_OXA-488_ showed higher prevalence in ICUs (22.4%, 19/85). This ICU-specific distribution suggests possible links to intensive care exposure factors.

**TABLE 2:** Drug-resistant genes carried by CRKP from different specimen types

Genomic features	Specimen types								*H*	*P*
	Blood (*n* = 5)	Bronchoalveolar lavage (*n* = 42)	Catheter (*n* = 2)	Others (*n* = 7)	Respiratory specimens (*n* = 57)	Sterile body fluids (*n* = 7)	Urine (*n* = 5)	Wound pus (*n* = 12)		
Drug-resistant genes M (P25, P75)	7 (7, 8)	7 (6, 7)	6 (6, 7)	7 (6, 7)	7 (6, 7)	7 (7, 7)	7 (6, 17)	6 (6, 7)	4.53	0.72

**TABLE 3:** Drug-resistant genes carried by CRKP from different departments (*n*, %)

β-Lactamase gene	ICUs (*n* = 85)	Non-ICU wards (*n* = 52)	*P*	*χ* ^2^
*bla*_OXA-396_	29 (34.1)	14 (26.9)	0.49	0.48
*bla*_OXA-50_	17 (20.0)	11 (21.2)	1.00	0
*bla*_OXA-488_	19 (22.4)	5 (9.6)	0.10	2.80
*bla*_OXA-395_	13 (15.3)	10 (19.2)	0.72	0.13
*bla*_OXA-486_	6 (7.1)	8 (15.4)	0.20	1.62
*bla*_OXA-10_	7 (8.2)	0 (0)	0.04^*a*^	–^*b*^
*bla*_VIM-2_	7 (8.2)	0 (0)	0.04^*a*^	–
*bla*_OXA-485_	1 (1.2)	4 (7.7)	0.07	–

^*a*^ *P *< 0.05.

^*b*^ “–,” not applicable.

Four plasmid replicon types were detected among 17 CRPA isolates: ColRNAI (*n* = 4), IncP-6 (*n* = 6), IncQ-2 (*n* = 8), and IncFII (*n* = 1; Table 4). ARGs were only identified in IncP-6 and IncQ-2 plasmids, with no resistance determinants found in ColRNAI or IncFII (Fig. 3B; Table S3). The resistance genes detected on the IncP-6 plasmid include *ant(3'')-Ia*, *bla*_OXA-10_, *qnrVC1*, *aac(6')-Ib-cr*, *aph(3')-VIb*, *sul1*, *ARR-3*, *dfrB4*, *qnrVC6*, and *bla*_VIM-2_. Moreover, six IncP-6-positive isolates within the same phylogenetic clade exhibited ≥2- to 32-fold higher resistance to 9/12 antibiotics (*P* < 0.05), with no difference to imipenem (*P* = 0.22) and meropenem (*P* = 0.09).

**TABLE 4:** Seventeen CRPA isolates carrying plasmid sequences

Number	ColRNAI_1	IncP6_1	IncQ2_1	IncFII(pHN7A8) _1
ly004	0	1	0	0
ly006	0	0	1	0
ly021	0	0	1	0
ly028	0	0	1	0
ly053	0	1	0	0
ly062	0	0	1	0
ly065	2	0	0	0
ly069	2	0	1	0
ly089	0	0	1	0
ly091	2	0	0	0
ly094	0	0	1	0
ly105	0	0	1	0
ly137	0	1	0	0
ly144	0	1	0	0
ly147	0	1	0	0
ly074	2	0	0	1
ly146	0	1	0	0

### Hospital transmission analysis

**Fig 4:**
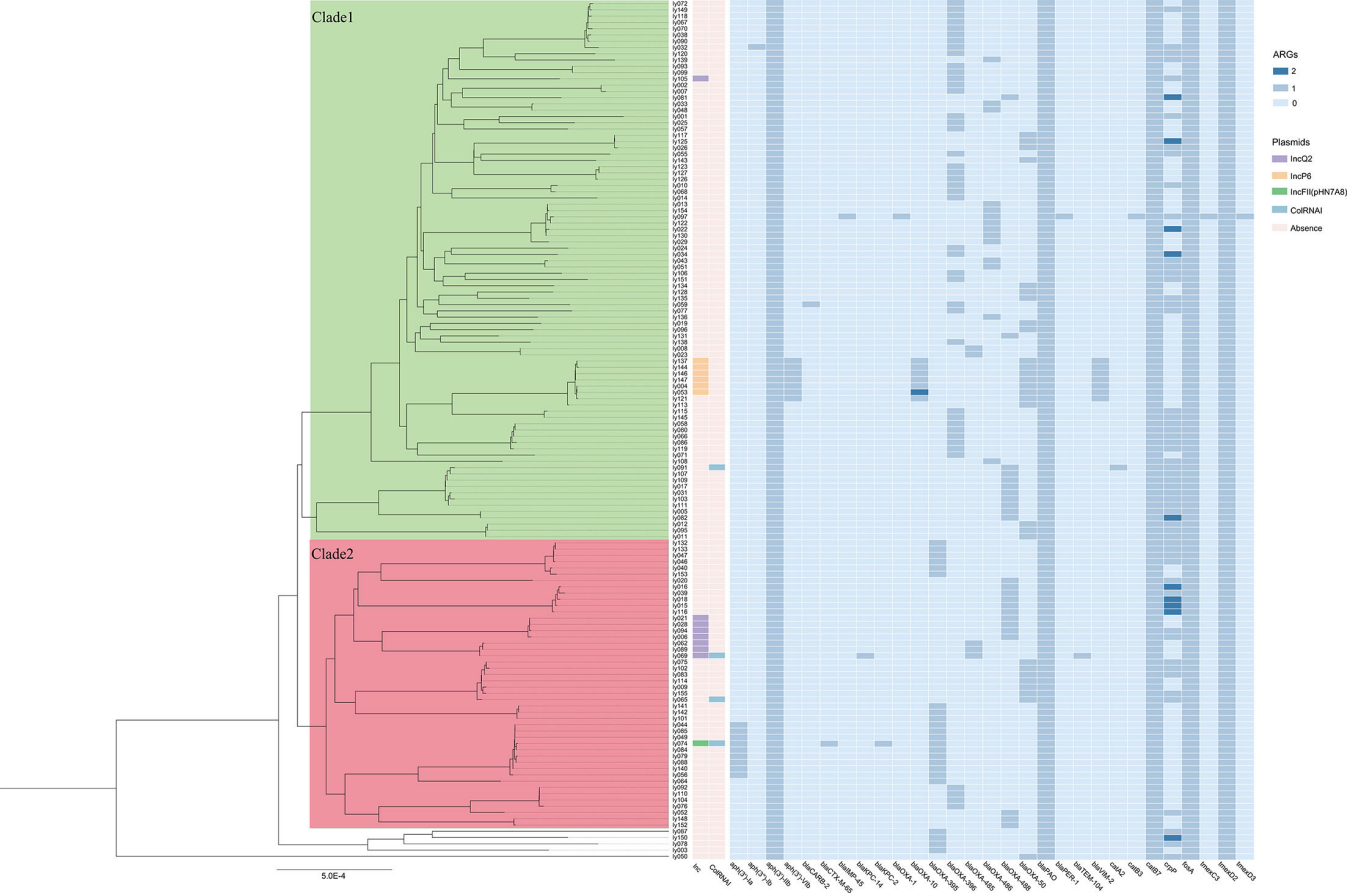
The phylogenetic tree and ARGs heatmap of 137 CRPA isolates. The phylogenetic tree based on the non-recombination alignment was constructed, with clades defined as 1 and 2, including five outlying isolates. The heatmap of ARGs for 137 CRPA isolates uses medium blue and dark blue squares to represent the presence of 1 and 2 corresponding ARGs, respectively.

**Fig 5:**
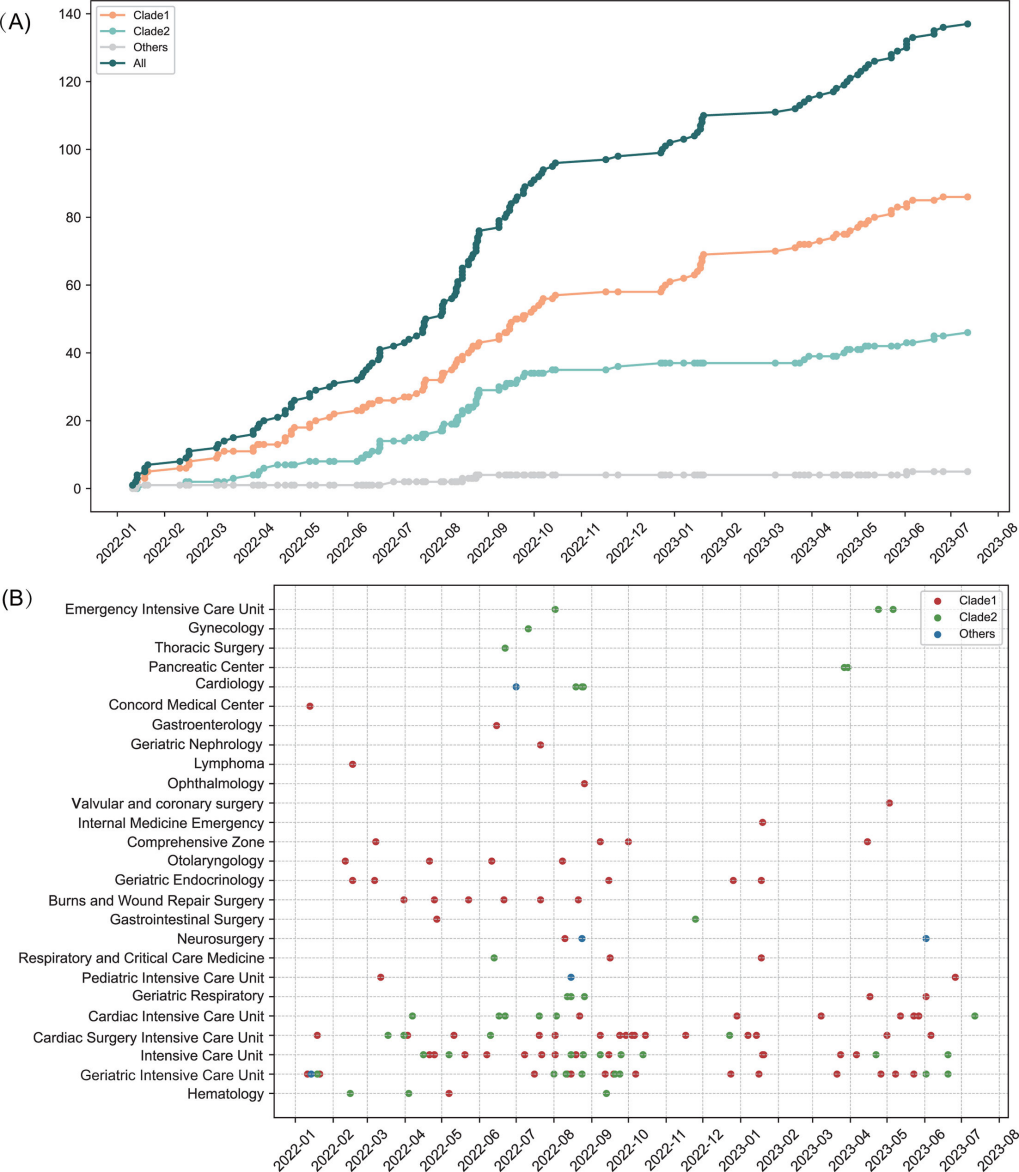
Persistence and co-occurrence of two distinct lineages (clade One and clade 2). (**A**) Cumulative distribution of CRPA isolates from January 2022 to July 2023, with data categorized by ALL (all isolates), clade 1, clade 2, and others. (**B**) Distribution of CRPA isolates across various departments from January 2022 to July 2023, categorized by clades: clade 1 (red), clade 2 (green), and others (blue).

One hundred thirty-seven CRPA isolates were sequenced using Illumina short reads and were *de novo* assembled. Mapping to the reference (GenBank: 2021CK-01227) showed 94% average coverage. A reference-based whole-genome alignment was constructed, from which 341,049 SNPs were identified. The pairwise disparity between isolates ranged from 0 to 93,759 SNPs. Putative recombination loci were further detected and removed, and 2,298 variable SNPs were identified. Phylogenetic analyses resolved the isolates into two major clades, together with five isolates falling outside the clades (Fig. 4). Two distinct transmission clusters were identified, corresponding to 86 isolates from clade 1 and 46 isolates from clade 2. Pairs of genomes within a distance of less than 25 SNPs were considered the same transmission cluster (Fig. S1 and S2). The pairwise number of SNPs within clade 1 isolates ranged from 0 to 254 SNPs (median, 157), which was comparable to the variation seen in clade 2 (0–224 SNPs; median, 185); 166–282 SNPs (median, 224) were found between clade 1 and 2 isolates. Epidemiological analysis revealed that clade 1 and clade 2 isolates, first detected in January 2022, circulated concurrently with parallel temporal trends (*P* > 0.05; Fig. 5A). Although clade 2 isolates were numerically more common in ICUs (65.2%, 30/46) than in non-ICUs (34.8%, 16/46), the distribution did not differ significantly (*χ*^2^=0.30, *P* = 0.59). Similarly, clade 1 showed no significant difference between ICUs and non-ICUs (*χ*^2^=0.02, *P* = 0.89). The SNP distances among IncP-6-positive isolates (ly004, ly053, ly137, ly144, ly146, and ly147) ranged from 0 to 3. Ly004 (clade 1) was initially detected in the GICU and later found in other departments, suggesting the GICU as a potential transmission hub (Fig. 5B). Using epidemiological data, we were able to confirm a significant spatiotemporal cluster in the GICU from 11 to 15 August 2022 (relative risk [RR] = 21.39, *P* = 0.01). An overlapping, higher-intensity sub-cluster was detected on 11 and 12 August (RR = 40.11, *P* = 0.02). The extreme RR values suggest transmission events: an acute point-source exposure and ongoing department-based transmission lasting through 15 August.

## DISCUSSION

The World Health Organization has classified CRPA as a “high-priority” pathogen requiring urgent epidemiological studies, enhanced surveillance, and novel antimicrobial development (23). A systematic review reported that ICU stay was one of the most significant risk factors for the acquisition of *P. aeruginosa* (24). Consistent with known high-risk settings (25), our CRPA isolates predominantly originated from ICUs and surgical departments (72.3%, 99/137), where critically ill patients requiring frequent invasive procedures and antibiotics face elevated infection risks. In this study, CRPA isolates were predominantly isolated from respiratory specimens and bronchoalveolar lavage, contrasting with findings from prior studies in Cameroon and Ethiopia (26, 27), while elderly male patients were the most common hosts.

*P. aeruginosa* infections have periodically increased in China, and the pathogen has shown a persistent resistance to commonly used antimicrobials (28). Our clinical CRPA isolates demonstrated high resistance rates to multiple anti-pseudomonal agents, aligning with recent CHINET surveillance data (http://www.chinets.com). However, the low resistance rates to aminoglycosides and polymyxin B can be attributed to their relatively high nephrotoxicity and pharmacokinetic issues, coupled with their less frequent use in clinical practice (29), resulting in lower resistance rates, which is consistent with the expected outcomes.

The detection of 54 STs in our study demonstrates the high genetic heterogeneity of CRPA isolates, consistent with findings from multiple regional surveillance studies (30, 31). Notably, our study identified several internationally recognized high-risk clones (ST244, ST277, and ST357) along with ST1971, a lineage reportedly prevalent in China (32, 33). Currently, a total of 20 O-serogroups were identified, with O1, O2, O5, O16, O6, and O11 responsible for 70% of *P. aeruginosa* infections (34). Notably, 66.4% of isolates in this study belonged to these high-risk O-serogroups, underscoring their public health significance.

The OXA-51-like subfamily is highly prevalent in *P. aeruginosa*, and previous studies suggest that the coexistence of *bla*_OXA-50_ and *crpP* contributes to the development of DTRPA, unlike the presence of *bla*_OXA-50_ alone (29). Although the *crpP* gene presence does not always correlate with ciprofloxacin resistance, the hypothesis that *crpP* functions as a ciprofloxacin kinase remains unchallenged (35). β-lactamases, including ESBLs, carbapenemases (metallo-β-lactamases [MBLs]), and AmpC cephalosporinases, represent a key resistance mechanism in *P. aeruginosa* (36), and plasmids are the main shuttles for ARG dissemination (37). Different plasmids carrying CR genes were reported in CRPA isolates (e.g., IncP-1, IncP-2, and IncP-6) (38, 39). The carbapenem resistance gene *bla*_KPC-2_ has been previously documented on IncP-6 plasmids in *P. aeruginosa* isolates (40). Here, an IncP-6 plasmid carrying *bla*_VIM-2_ was identified in CRPA for the first time. Our data highlight ICU environments as critical reservoirs for high-risk resistance genes. The exclusive detection of *bla*_OXA-10_ and *bla*_VIM-2_ in ICU isolates likely reflects intense antibiotic selection pressure, particularly from extended-spectrum cephalosporins and carbapenems. Although both genes are located on the IncP-6 plasmid, their restriction to isolates of the same ST suggests clonal expansion of a bacterial lineage carrying this plasmid. Moreover, integration of these ARG-containing accessory genetic elements may promote CRPA adaptation and persistence in hospital environments under antimicrobial selection pressure (41). Continuous surveillance of the prevalence of plasmids is essential to evaluate their potential role in enhancing the transmissibility of pathogens and to track their spread across different departments.

Phylogenetic and epidemiological analyses revealed distinct transmission dynamics of the target pathogen, with two predominant clades identified (clade 1, *n* = 86; clade 2, *n* = 46). While temporal analysis showed parallel circulation patterns (*P* > 0.05), significant genomic divergence between clades suggested separate introduction events. Genomic analysis detected a cluster of IncP-6-positive isolates demonstrating high homology (0–3 SNPs), with epidemiological evidence suggesting the GICU as the probable origin, followed by dissemination to other departments. Spatiotemporal analysis further confirmed a high-risk transmission event in GICU (11–15 August 2022; RR = 21.39–40.11), indicating a point-source exposure followed by nosocomial spread. Our findings align with prior studies highlighting ICUs as hubs for resistant or highly transmissible pathogens (42, 43). However, we observed no statistically significant ICU predominance for clades, possibly due to limited sample size or shared transmission routes between clades. The parallel circulation of clade 1 and clade 2 further suggests multiple independent transmission chains, warranting validation via epidemiological data (e.g., patient transfer records). To mitigate the environmental spread of resistant bacteria, it is crucial to implement enhanced disinfection protocols, establish genomic surveillance networks for cross-institutional tracking, and adopt targeted interventions such as pre-ICU decolonization, which has been shown to reduce ICU-acquired infections (44, 45).

Our study had several limitations. (i) This study did not collect clinical epidemiological data (e.g., invasive procedures, antibiotic exposure, or patient outcomes). Future studies combining microbiological and clinical data are needed to validate these associations. (ii) Although preliminary data indicate potential differences in resistance gene profiles, the limited sample size may result in low statistical power, underscoring the necessity of validation in larger cohorts. (iii) Our study lacks patient movement data between departments, shared equipment logs, or healthcare worker rotation data, which limit our ability to definitively explain how the isolates spread between units. Genomic clustering should be interpreted alongside clinical epidemiology to infer origins.

## Data Availability

WGS were deposited in National Microbiology Data Center (NMDC) with accession number NMDC10018733.

## SUPPLEMENTAL MATERIAL

Supplemental Figures spectrum.00433-25-s0001.docx Fig. S1 and S2.

Supplemental Tables spectrum.00433-25-s0002.xlsx Tables S1 to S4.

ASM does not own the copyrights to Supplemental Material that may be linked to, or accessed through, an article. The authors have granted ASM a non-exclusive, world-wide license to publish the Supplemental Material files. Please contact the corresponding author directly for reuse.
