# Supplementary material for: Molecular insights into the persistence and co-occurrence of two different carbapenem-resistant Pseudomonas aeruginosa lineages within a hospital setting
Source: Microbiol Spectr. 2025 Sep 17;13(10):e00433-25. doi: 10.1128/spectrum.00433-25 (PMC12502679; doi:10.1128/spectrum.00433-25)
Supplement: Supplemental Figures — Fig. S1 and S2. [file spectrum.00433-25-s0001.docx]

**Supplementary figures**


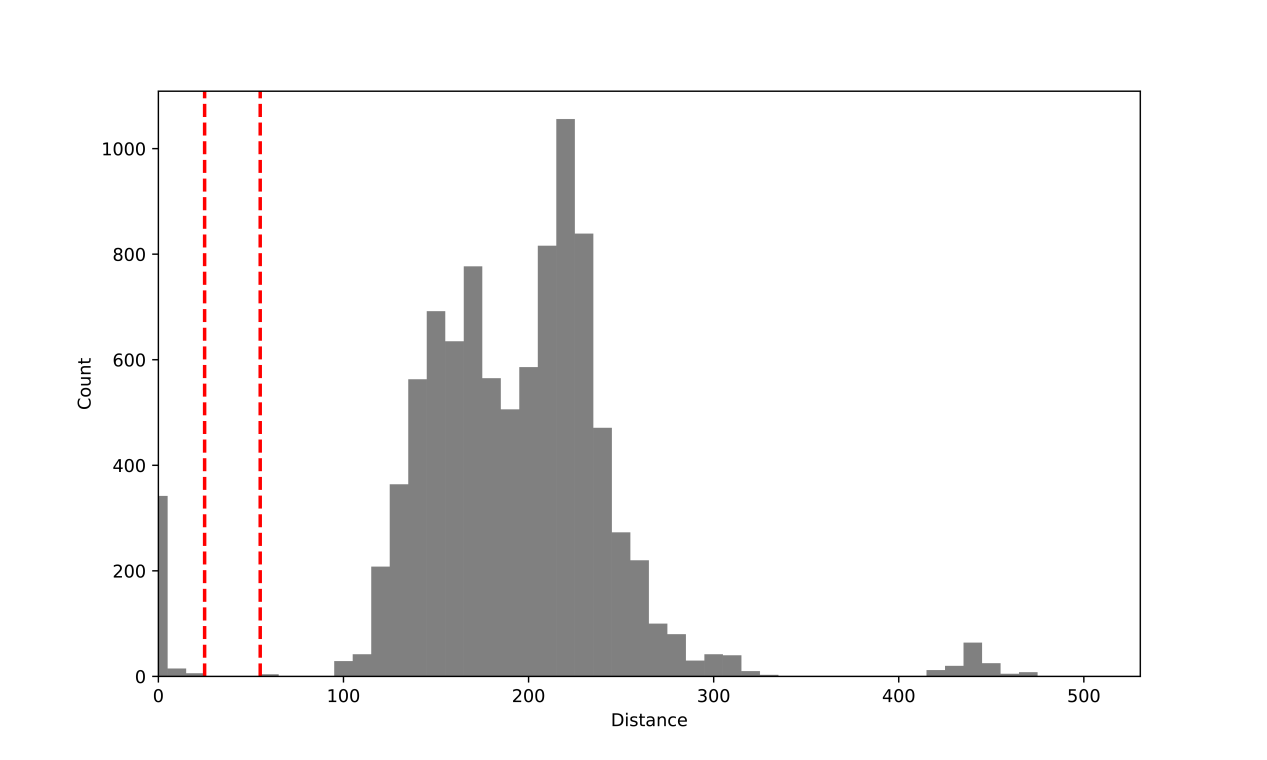


Figure S1

Genome pairs with ≤ 25 SNP differences were classified as outbreak-associated.
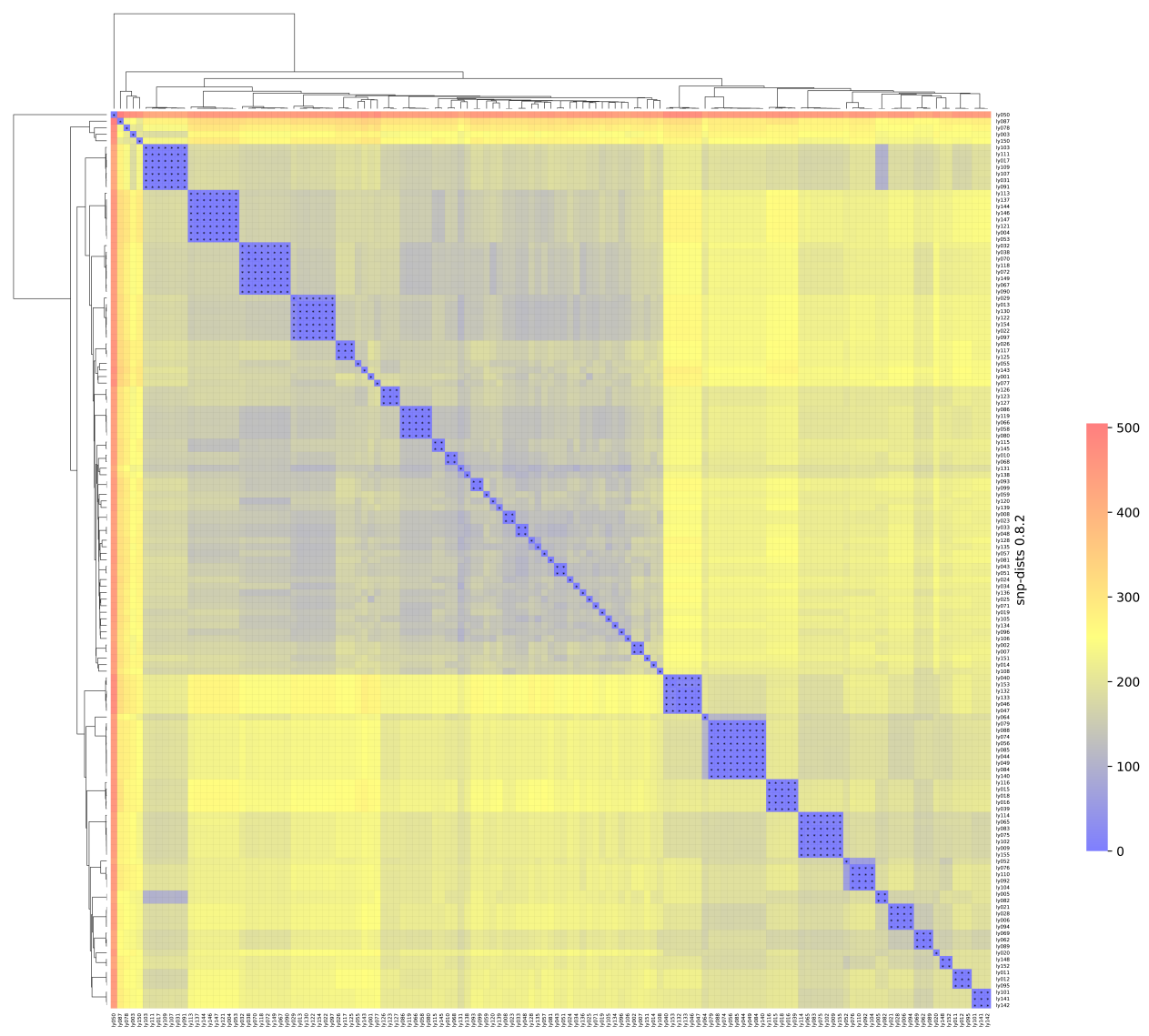


Figure S2

A matrix of pairwise SNP distances between CRPA isolates, with values color-coded according to the scale (right). Genome pairs differing by ≤ 25 SNPs were marked with asterisks.
